# Portion size and consistency as indicators of complementary food energy intake

**DOI:** 10.1111/mcn.13121

**Published:** 2021-02-02

**Authors:** Emily C. Faerber, Aryeh D. Stein, Amy Webb Girard

**Affiliations:** ^1^ Hubert Department of Global Health, Rollins School of Public Health Emory University Atlanta Georgia USA

**Keywords:** complementary feeding, dietary assessment tools, dietary intake assessment, indicator development, infant and child nutrition, survey methods

## Abstract

We evaluated whether novel portion size and consistency indicators can identify children with low complementary food energy intake in southern Ethiopia. We conducted 24‐h dietary recalls with caregivers of 548 children aged 6–13 months; additionally, caregivers estimated their child's usual portion size using uncooked rice and selected which of five photographs of porridges of varying consistencies most closely matched the food their child usually ate. Complementary food energy and density from the 24‐h recall were used as reference values. We computed correlation coefficients and areas under receiver operating characteristic curves (AUC) and conducted sensitivity and specificity analyses to classify children with low complementary food energy intake. The median complementary food energy intakes for children 6–8, 9–11 and 12–13 months were 312, 322 and 375 kcal; median estimated portion sizes were 50, 58 and 64 ml, respectively. Estimated portion size correlated with total complementary food energy intake and with average energy and quantity consumed per feeding (*r* = 0.42, 0.46 and 0.45, respectively, all *p* < 0.001). Reported food consistency was weakly correlated with total complementary food energy intake (*r* = 0.18) and density (*r* = 0.10), and energy density of porridge only (*r* = 0.24, all *p* < 0.05). Predicted energy intake combining feeding frequency and portion size predicted inadequate energy intake better than did feeding frequency alone in infants 6–8 months [∆AUC = 0.16, 95% confidence interval (CI) 0.04, 0.28] and 9–11 months (∆AUC = 0.09, 95% CI 0.04, 0.14). Caregiver estimates of portion size can improve identification of infants with low complementary food energy intake when more robust dietary assessment is not feasible.

**Key messages**

- Energy intake from complementary foods is a function of feeding frequency, energy density of complementary foods and amount consumed per feeding.
- Caregivers' estimate of usual portion size using uncooked rice is correlated with complementary food intake and energy intake.
- A five‐photograph indicator of complementary food consistency is weakly correlated with complementary food energy intake and energy density.
- Portion size and feeding frequency combined better predict young children with inadequate energy intake from complementary foods, compared with feeding frequency alone.

## 1 INTRODUCTION

Complementary food energy intake is a function of feeding frequency, amount consumed per feeding, and energy density (PAHO/WHO, 2003; World Health Organization, 1998). The most precise methods for estimating energy intake are expensive, time‐consuming, require skilled data collectors and impose a high respondent burden. Estimating intake of infants and young children in low‐ and middle‐income countries is especially challenging (Foster & Adamson, 2014; Paintal & Aguayo, 2016; Quandt, 1987; Shankar et al., 2001). The World Health Organization (WHO) developed indicators of infant and young child feeding (IYCF) to enable population‐level IYCF assessment for monitoring, evaluation and research (Ruel, Brown, & Caulfield, 2003) where more robust methods of assessment are resource‐prohibitive. The WHO IYCF indicator definitions were published in 2008, with an ensuing implementation guide in 2010 (WHO, 2008, 2010). The indicators have since been widely adopted but are limited in their scope (Ruel, 2017).

Feeding frequency, the number of meals and snacks consumed in a 24‐h period, was previously evaluated as an indicator of energy intake using 10 data sets from nine countries in Africa, Asia and Latin America (Working Group on Infant and Young Child Feeding Indicators, 2006, 2007) and is currently the only IYCF indicator of complementary food energy intake. Portion size and energy density are difficult to estimate using questionnaire‐based approaches. Weighing food is the gold standard for measuring intake but is impractical for large surveys. A number of portion size estimation aids have been developed and evaluated, including food photographs (Amougou et al., 2016; Bouchoucha et al., 2016; Flax et al., 2019; Foster et al., 2017; Harris‐Fry et al., 2016; Huybregts, Roberfroid, Lachat, Van Camp, & Kolsteren, 2008; Korkalo, Erkkola, Fidalgo, Nevalainen, & Mutanen, 2013; Lazarte, Encinas, Alegre, & Granfeldt, 2012; Naska et al., 2016; Nichelle et al., 2019; Nissinen et al., 2018; Tueni, Mounayar, & Birlouez‐Aragon, 2012; Turconi et al., 2005; Venter, MacIntyre, & Vorster, 2000), playdough or uncooked rice (Nightingale et al., 2016), and a cube system called the ‘International Food Unit’ (Bucher et al., 2017). However, the validity of these portion size estimation aids has only been assessed for estimating portions of a specific food at a specific meal and/or in the context of dietary recall (such as 24‐h recall or food frequency questionnaires). However, there are no validated methods for assessing *average* or *usual* portion size. Complementary food consistency has been used as a proxy of energy density. Detailed methods for assessing complementary food consistency in surveys are often not described in detail, but results have been reported as adjectives such as *liquid/gruel‐like/solid* (Chapagain, 2013; Chauhan, Bala, Nandan, & Misra, 2007; Kamenju et al., 2016; Kamenju et al., 2017; Mishra, Kumar, Basu, Rai, & Aneja, 2014; Oliveira, Castro, & Jaime, 2014). However, in southern Ethiopia, caregivers verbally overestimated complementary food thickness compared with observed consistencies (Alive & Thrive, 2010), suggesting that use of descriptive adjectives may be unreliable. Aggarwal, Verma, Faridi and Dayachand (2008) used drawings from a WHO training slide to assess consistency of complementary foods, whereas Jones (2015) used photographs of porridges of varying consistencies in the context of a 24‐h dietary recall.

The objective of the present research was to estimate the added value of low‐burden field methods for indicators of portion size and complementary food consistency when screening children at risk of low complementary food energy intake in a sample of children 6–13 months old in southern Ethiopia.

## 2 METHODS

### 2.1 Study setting and population

Data for this study were collected as part of a cluster‐randomized nutrition‐sensitive agriculture project evaluation in the Sidama and Gedeo zones in the Southern Nations, Nationalities, and Peoples' Region (SNNPR), Ethiopia. The trial consisted of a control group (*n* = 235), a partial intervention group (*n* = 143) and a full intervention group (*n* = 170). Both intervention groups received agricultural inputs and support for orange‐fleshed sweet potato agriculture as well as community‐based nutrition education that includes messaging on age‐appropriate portion size and complementary food consistency. The full intervention group also received a feeding bowl and spoon designed to promote age‐appropriate portion sizes and complementary food consistency (Collison et al., 2015; Kram et al., 2016).

### 2.2 Data collection

Caregivers with infants less than 6 months completed a baseline survey in January 2018, immediately prior to the initiation of community‐based nutrition education in intervention communities. Households were considered eligible if they had an infant under 6 months without a serious health condition and (for intervention groups) if they were enrolled in implementation activities. A sample size of 600 households was sought at the baseline survey for adequate power to detect increases in energy intake of 100–150 kcal. Households completed a follow‐up survey in August 2018; at this time, households in the intervention group were nearing their ‘graduation’ from intervention activities, though activities were being scaled to other households within those communities. Because infants were all under 6 months at baseline, only data from follow‐up are used in these analyses. The exception is caregiver education, which was only assessed at baseline.

At follow‐up, all infants were at least 6 months of age, but not more than 13 months. Therefore, we group the sample by the following age groups: 6–8, 9–11 and 12–13 months. The follow‐up survey included WHO IYCF indicators (WHO, 2008, 2010), novel indicators of usual portion size and complementary food consistency, and 24‐h dietary recall of infant diets using the methodology described by Gibson and Ferguson (2008). Food composition, recipe and conversion factor databases were developed using primary data collection and/or pre‐existing data from Uganda (Hotz, Abdelrahman, Sison, Moursi, & Loechl, 2012), Ethiopia (Ethiopian Health and Nutrition Research Institute, 1968–1997) or, where necessary, the United States Department of Agriculture (U.S. Department of Agriculture, 2019).

Survey enumerators with university education, who spoke English, Amharic and at least one of the two local languages of the study area underwent a 1‐week training prior to the baseline survey and a 2‐week training prior to the follow‐up survey. Training included conducting pilot surveys. Data were collected on paper questionnaires. Survey data were then doubly entered into CSPro 7.1 with any discrepancies resolved by a survey supervisor or member of the research team. Data from the multiple‐pass 24‐h dietary recall were entered into CSDietary software (HarvestPlus, 2020), with a member of the research team then visually inspecting to verify the accuracy of data entry.

Written informed consent was obtained from caregivers prior to each survey, and enumerators received training in research ethics.

### 2.3 Reference intakes

Multiple‐pass 24‐h dietary recall was used to estimate total energy intake from complementary foods, average energy intake per feeding, average quantity (in grams) of complementary food consumed per feeding, average energy density of complementary foods and average energy density of porridges/gruels. If a child consumed no complementary food or liquid (other than water), then, his or her energy intake was set to 0 kcal, but energy density was considered missing. Thresholds for classifying children as having low complementary food energy intake were as follows: <202 kcal for 6 to 8 months, <307 kcal for 9 to 11 months, and <548 kcal for 12 months and older (Dewey & Brown, 2003).

### 2.4 Test indicators

**FIGURE 1:**
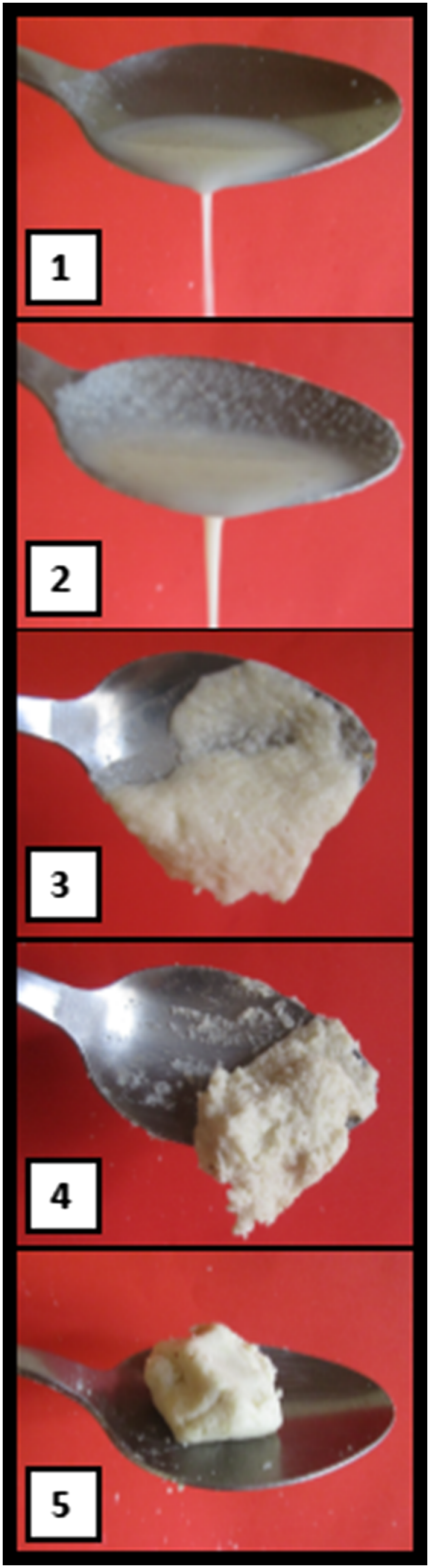
Photographs of porridge with energy densities (1) 0.1, (2) 0.4, (3) 0.7, (4) 0.9 and (5) 1.3 kcal/g, used to assess usual complementary food consistency in a household survey in Southern Nations, Nationalities, and Peoples' Region (SNNPR), Ethiopia

To estimate portion size, caregivers were first asked whether their child normally eats from a shared dish or receives his or her own dish. For children who receive their own dish, caregivers used uncooked rice to estimate a *typical* portion served to the child; this volume of uncooked rice was then transferred to a graduated cylinder and the volume recorded to the nearest millilitre (ml). The caregiver was asked if the child typically leaves any food remaining, and if so, was asked to use uncooked rice to estimate the amount of food uneaten, such that the amount consumed could be calculated as the volume remaining subtracted from the volume served. If the caregiver answered that the child typically eats from a shared dish, then the caregiver used the uncooked rice to estimate the amount consumed. To estimate complementary food consistency, caregivers were shown five numbered photographs of porridges with varying consistencies (Figure 1) and were asked to select which photograph most closely resembled the consistency of complementary foods eaten by their child. The five photographs depicted porridges with energy densities of 0.1, 0.4, 0.7, 0.9 and 1.3 kcal/g, respectively. Each photograph depicts a porridge prepared with maize flour and vegetable oil, which are locally available and were identified through formative research as common ingredients for porridge. Each ingredient was weighed, mixed with boiling water and stirred, then allowed to cool for 5 min before the final porridge was weighed (so that the energy density could be calculated) and photographed. These methods of assessing portion size and complementary food consistency are considered test indicators.

We computed predicted energy intake (PEI) as the product of portion size (millilitre per feeding) and feeding frequency (feedings per day), while assuming an energy density of 1.0 kcal/g and a complementary food density of 1.05 g/ml, which is the density of porridge prepared with maize flour according to the INFOODS Density Database Version 2.0 (FAO/INFOODS, 2012). We also computed a modified predicted energy intake (PEI‐M) as the product of portion size (millilitre per feeding), feeding frequency (feedings per day) and the energy density of the photograph selected by the caregiver.

### 2.5 Analytical approach

We assessed the distributions of continuous variables for normality based on skewness, kurtosis and visual inspection of histograms.

We computed correlation coefficients between references and test indicators. For portion size, references were total energy intake from complementary food, average energy intake per feeding and average amount of complementary food, in grams, consumed per feeding. For consistency, the references were total energy intake from complementary foods, average energy density of complementary foods and average energy density of porridges/gruels. We also assessed the correlations between feeding frequency and total energy intake from complementary foods and number of feeding episodes reported during the 24‐h recall.

We used Pearson's correlation coefficients for normally distributed continuous variables and Spearman's rank correlation coefficients for non‐normally distributed variables. Feeding frequency was treated as a continuous variable. Consistency was treated as an ordinal variable, for which we used polyserial correlation coefficients.

We computed correlations for the full sample and for subgroups of children whose caregiver reported that the child had consumed solid, semi‐solid or soft foods in the previous day; by age category (6 to 8, 9 to 11 and 12–13 months); whether the child eats from a shared dish versus his/her own dish; by intervention group; by Sidama versus Gedeo zone; whether or not the child had been sick in the previous day; whether or not anyone in the household had fasted in the previous day; and whether or not the caregiver had completed primary school. We used Fisher's *r*‐to‐*z*‐transformation to test whether correlation coefficients differed between these strata.

Using the conversion of 1.05 g/ml (FAO/INFOODS, 2012), we converted caregivers' estimates of usual portion size to grams to assess agreement between estimated portion size and average amount of food consumed per episode, both in grams, using a Bland–Altman plot.

We computed sensitivity, specificity and area under the receiver operating characteristic (ROC) curve (AUC) to assess the ability of the indicators to identify children whose total complementary food energy intake, as measured by 24‐h recall, was low. For the sensitivity and specificity of portion size, we used 10‐ml increments. We used the ROCCONTRAST statement in SAS 9.4 (SAS Institute Inc., Cary, NC, USA) to test whether any of the indicators' AUC was significantly different from feeding frequency. We then classified children as either at risk or not at risk of low complementary food energy intake based on whether the child met thresholds for portion size and consistency that were identified through sensitivity and specificity analyses. Using this dichotomous classification, we computed sensitivity and specificity for identifying children with low complementary food energy intake. Sensitivity, specificity and ROC curves were estimated separately for each age group (6–8, 9–11 and 12–13 months).

A *p* value less than 0.05 was considered statistically significant. All analyses were conducted in SAS 9.4 (SAS Institute Inc., Cary, NC, USA).

### 2.6 Ethical considerations

Ethical approval for this work was obtained from Emory University's Institutional Review Board, and from the Southern Nations, Nationalities and Peoples' Regional Bureau of Health Ethical Review Committee. The trial for which the data were collected is registered with ClinicalTrials.gov, ID NCT03423472.

## 3 RESULTS

In total, 548 caregiver–child dyads completed the follow‐up survey and were eligible for the analyses described here. General characteristics of the sample are shown in Table 1. Children ranged in age from 6 to 13 months (mean 10.0 ± 1.7 months), and 18.3% were reportedly sick in the previous day. A minority (6.4%) of households had a member who had fasted in the previous day, and 18.1% of caregivers had completed primary school. Only one child was not breastfed, and 96.2% of caregivers said their child received ‘solid, semi‐solid or soft foods’ in the previous day. The mean number of feeding episodes was 3.6 ± 1.7. A majority of caregivers (87.3%) reported that the child typically receives his or her own feeding dish. Most children (65.1%) reportedly consume complementary food of medium consistency, matching the third photograph (0.7 kcal/g); almost 20% of children consumed thin complementary foods (photograph 1 or 2), and just over 15% consumed thicker complementary foods (photograph 4 or 5).

**TABLE 1:** Characteristics and feeding practices of young children 6–13 months at follow‐up, residing in Southern Nations, Nationalities, and Peoples' Region (SNNPR), Ethiopia, overall and by age category

Characteristic	Total	6–8 months	9–11 months	12–13 months
	(*N* = 548)	(*n* = 125)	(*n* = 307)	(*n* = 116)
Age (months)	10.0 ± 1.7	7.5 ± 0.6	10.1 ± 0.8	12.2 ± 0.5
Female	50.0	50.4	49.8	50.0
Sick in previous day	18.3	19.2	19.9	12.9
Zone				
Sidama	40.9	40.0	39.1	46.6
Gedeo	59.1	60.0	60.9	53.5
Anyone in household fasted in previous day	6.4	8.8	4.9	7.8
Caregiver completed primary school^a^	18.1	19.5	19.9	12.1
Infant and young child feeding practices^b^				
Breastfed	99.8	100.0	99.7	100.0
Received solid, semi‐solid or soft foods	96.2	96.8	94.8	99.1
Feeding frequency	3.6 ± 1.7	3.4 ± 1.5	3.5 ± 1.8	3.8 ± 1.7
Consistency				
Photograph 1 (thinnest)	2.4	4.3	2.2	0.9
Photograph 2	17.1	25.0	16.3	10.9
Photograph 3	65.1	59.5	65.6	70.0
Photograph 4	11.6	9.5	10.9	15.5
Photograph 5 (thickest)	3.8	1.7	5.1	2.7
Receives own feeding dish	87.2	90.2	83.3	94.0
Portion size, ml [median (IQR)]	55 (40, 80)	50 (36, 70)	58 (39, 80)	64 (45, 80)
Estimated nutrient intake from complementary foods				
Energy intake, kcal [median (IQR)]	331 (190, 490)	312 (172, 447)	322 (187, 502)	375 (250, 571)
Adequate energy from complementary foods^c^	50.7	68.8	52.4	26.7
Energy density, kcal/g [median (IQR)]	1.5 (1.2, 1.9)	1.6 (1.2, 1.9)	1.5 (1.2, 1.9)	1.6 (1.3, 1.9)
Adequate energy density of complementary foods^d^	95.5	97.5	94.7	95.7

*Note*. Values are mean ± standard deviation or percentage, unless otherwise noted.

Abbreviation: IQR, interquartile range; SD, standard deviation.

^a^ Caregiver education was assessed only at the baseline survey.

^b^ Breastfeeding status, receipt of solid, semi‐solid or soft foods, and feeding frequency are based on recall of previous day using World Health Organization methodology [CITATION], whereas receipt of own feeding dish, consistency and portion size are based on usual practice as reported by a caregiver.

^c^ Energy intakes greater than or equal to 202, 307 and 548 kcal for children ages 6–8, 9–11 and 12–13 months, respectively, are considered adequate.

^d^ An average energy density greater than or equal to 0.8 kcal/g is considered adequate.

The median [interquartile range (IQR)] portion size for infants 6–8 months was 50 (IQR 35, 70) ml; for infants 9–11 months, it was 58 (IQR 39, 80) ml and for children over 12 months, it was 64 (IQR 45, 80) ml (*n* = 125, 307 and 116, respectively). Just over half (50.7%) of the sample had adequate energy intake from complementary foods, but this proportion was highest in infants 6–8 months (68.8%) and lowest in children over 12 months (26.7%). The median energy density of complementary foods was 1.5 (IQR 1.2, 1.9) kcal/g, and a high proportion (95.5%) consumed complementary foods with an average energy density of at least 0.8 kcal/g (PAHO/WHO, 2003). Portion size, energy intake from complementary foods, average energy intake per feeding episode, amount of food consumed per eating episode and energy density were all right‐skewed.

Estimated portion size correlated positively with total energy intake from complementary foods (*r* = 0.42), average energy intake per feeding episode (*r* = 0.46) and average quantity consumed per feeding episode (*r* = 0.45, all *p* < 0.001; Table 2). Despite some heterogeneity in the strength, portion size correlations were significant in every subgroup with the exception of the correlation with total complementary food energy intake among children who had been sick in the previous day, where the sample size was only 97 children (*r* = 0.14, *p* > 0.05). The Bland–Altman plot revealed that portion size estimates became less precise with increasing intake (Figure S1). The mean bias (estimated portion size minus average amount consumed per feeding episode) was 5.8 g (95% limits of agreement −60.3, 72.0 g).

**TABLE 2:**
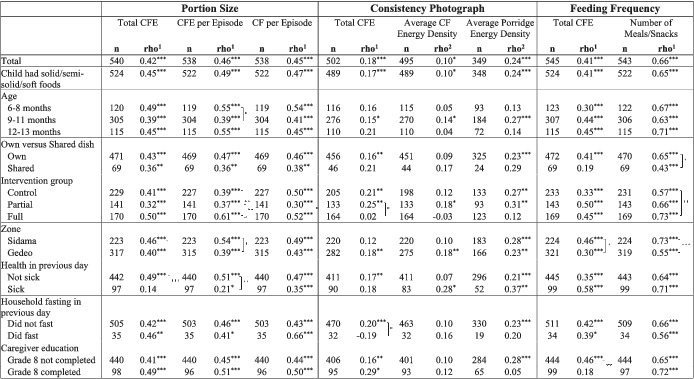
Correlation coefficients between the following: portion size indicator and total complementary food energy, complementary food energy per episode and quantity of complementary food consumed per episode; consistency indicator and total complementary food energy, average complementary food energy density and average porridge energy density; and feeding frequency indicator and total complementary food energy and number of meals/snacks reported in 24‐h recall, for 548 young children from SNNPR, Ethiopia

*Note*. Brackets show where correlations differ significantly by subgroup, based on Fisher's *r*‐to‐*z* transformation.

Abbreviations: CF, complementary food; CFE, complementary food energy; SNNPR, Southern Nations, Nationalities, and Peoples' Region.

^a^ Spearman's rank correlation coefficients.

^b^ Polyserial correlation coefficients.

* *p* < 0.05.

** *p* < 0.01.

*** *p* < 0.001.

The complementary food consistency photograph number was weakly correlated with total complementary food energy intake (*r* = 0.18, *p* < 0.001), average energy density of complementary foods (*r* = 0.10, *p* < 0.05) and average energy density of only porridges/gruels (*r* = 0.24, *p* < 0.001). Correlation of the consistency photograph number with total complementary food energy was significantly greater in the partial (*n* = 164) compared with the full intervention group (*n* = 133), and among households in which no member fasted in the previous day (*n* = 470) compared with those who did (*n* = 32).

Reported feeding frequency was significantly correlated with both total complementary food energy intake (*r* = 0.41, *p* < 0.001) and with actual number of feeding episodes as reported in the 24‐ dietary recall (*r* = 0.66, *p* < 0.001). The strength of the correlations differed in some subgroups, but with few exceptions where strata sample sizes were small, remained significant in every group.

Each of the individual indicators, as well as PEI and PEI‐M, significantly predicted low complementary food energy intake (Table 3). When compared with the AUC of feeding frequency, the AUC of portion size was similar in each age group, whereas the consistency indicator performed significantly worse among children 9–11 months (*n* = 276, ∆AUC = −0.12, 95% CI −0.20, −0.04) and 12–13 months (*n* = 110, ∆AUC = −0.19, 95% CI −0.32, −0.07). Both PEI and PEI‐M performed significantly better than feeding frequency among infants 6–8 months (*n* = 118, ∆AUC = 0.16, 95% CI 0.04, 0.28 and *n* = 112, ∆AUC = 0.21, 95% CI 0.10, 0.33, respectively) and 9–11 months (*n* = 305, ∆AUC = 0.09, 95% CI 0.04, 0.14 and *n* = 276, ∆AUC = 0.07, 95% CI 0.01, 0.13, respectively). Among children 12–13 months of age, the AUC of PEI and PEI‐M were similar to the AUC of feeding frequency alone.

**TABLE 3:** Areas under receiver operating characteristic curves for individual and combined complementary food energy indicators predicting low complementary food energy intake among 548 young children in SNNPR, Ethiopia, by age category

Age category	*n*	AUC	95% CI	∆AUC	95% CI
6–8 months (<202 kcal)					
Feeding frequency	123	0.65**	0.54, 0.76	Ref.	
Consistency	116	0.63**	0.54, 0.73	0.00	−0.16, 0.16
Portion size	120	0.72***	0.62, 0.83	0.10	−0.08, 0.28
PEI	118	0.78***	0.69, 0.88	0.16**	0.04, 0.28
PEI‐M	112	0.84***	0.76, 0.93	0.21***	0.10, 0.33
9–11 months (<307 kcal)					
Feeding frequency	307	0.69***	0.63, 0.74	Ref.	
Consistency	276	0.56*	0.50, 0.62	−0.12**	−0.20, −0.04
Portion size	305	0.69***	0.63, 0.75	0.00	−0.08, 0.09
PEI	305	0.78***	0.73, 0.83	0.09***	0.04, 0.14
PEI‐M	276	0.75***	0.70, 0.81	0.07*	0.01, 0.13
12–13 months (<548 kcal)					
Feeding frequency	115	0.79***	0.70, 0.87	Ref.	
Consistency	110	0.60*	0.51, 0.68	−0.19**	−0.32, −0.07
Portion size	115	0.73***	0.64, 0.83	−0.06	−0.19, 0.07
PEI	114	0.84***	0.77, 0.92	0.06	0.00, 0.12
PEI‐M	108	0.85***	0.78, 0.92	0.07	0.00, 0.13

Abbreviations: AUC, area under the curve; CI, confidence interval; PEI, predicted energy intake; PEI‐M, modified predicted energy intake; Ref, Reference; SNNPR, Southern Nations, Nationalities, and Peoples' Region.

* *p* < 0.05.

** *p* < 0.01.

*** *p* < 0.001.

Portion size cut‐offs of <50, <60 and <70 ml among children 6–8, 9–11 and 12–13 months, respectively, yielded sensitivities and specificities that were both at least 0.60 for classifying children with low complementary food energy intake (Table S1). No clear cut‐off in consistency was identified (Table S2). In each age group, the second photograph was highly specific (0.77–1.0) but not sensitive (0.16–0.43). However, the third photograph was sensitive (0.84–0.97) but not specific (0.15–0.23). The cut‐off of minimum meal frequency resulted in low sensitivity (0.18–0.33) and high specificity (0.88–1.0; Table 4).

**TABLE 4:** Sensitivity/specificity analysis of complementary feeding indicators for low complementary food energy intake for 548 young children in SNNPR, Ethiopia, by age category

Age category	*n*	Sensitivity	Specificity	PPV	NPV	% misclassified
6–8 months (<202 kcal)						
≤1 feeding episodes	123	0.18	0.99	0.88	0.72	26.8
Portion size (<50 ml)	120	0.77	0.62	0.46	0.87	33.3
≤photo 2	116	0.43	0.77	0.44	0.76	33.6
≤1 feeding episodes or <50 ml	118	0.86	0.61	0.48	0.91	31.4
≤1 feeding episodes or <50 ml or ≤photo 2	112	0.91	0.45	0.42	0.92	41.1
9–11 months (<307 kcal)						
≤2 feeding episodes	307	0.33	0.88	0.72	0.59	38.1
Portion size (<60 ml)	305	0.66	0.61	0.60	0.66	37.0
≤photo 2	276	0.23	0.85	0.55	0.58	42.4
≤2 feeding episodes or <60 ml	305	0.79	0.54	0.61	0.74	33.8
≤2 feeding episodes or <60 ml or photo 2	276	0.78	0.46	0.53	0.72	39.9
12–13 months (<548 kcal)						
≤2 feeding episodes	115	0.20	1.00	1.00	0.32	58.3
Portion size (<70 ml)	115	0.67	0.65	0.84	0.42	33.9
≤photo 2	110	0.16	1.00	1.00	0.31	60.9
≤2 feeding episodes or <70 ml	114	0.75	0.65	0.85	0.49	28.1
≤2 feeding episodes or <70 ml or ≤photo 2	108	0.76	0.63	0.84	0.50	27.8

Abbreviations: PPV, positive predictive value; NPV, negative predictive value; SNNPR, Southern Nations, Nationalities, and Peoples' Region.

A dichotomous indicator of whether children met both feeding frequency and portion size cut‐offs identified above resulted in higher sensitivity (0.75–0.86) but lower specificity (0.54–0.65) compared with individual indicators. The proportions of children in each age group who were misclassified using this dichotomous indicator ranged from 28.1% to 33.8%. The dichotomous indicator that also included having a consistency matching photograph 3 or higher had improved sensitivity (0.76–0.91) but lower specificity (0.45–0.63), and the proportions of children misclassified ranged from 27.8% to 41.1%.

## 4 DISCUSSION

We found that caregiver estimates of portion size were correlated with their recall of complementary food intake and energy intake and that when combined with minimum meal frequency, these estimates aided in the identification of children with low complementary food energy intake. A five‐photograph indicator of complementary food consistency added little to the ability to identify children with low complementary food energy intake.

The methods described and evaluated here provide a means to assess usual portion size and complementary food consistency as a proxy of energy density that would not require the resources of a more robust method of dietary assessment. Multiple‐pass 24‐h dietary recall, as described by Gibson and Ferguson (2008) is burdensome and expensive. Caregivers are asked to attend an introductory meeting 2 days before the recall, and based on our experience in this survey, the total time required for the caregiver training and dietary recall can exceed 2 h in addition to caregivers' travel time. Furthermore, dietary recall requires extensive formative research to understand details of the local diet for the development of probing questions for each food consumed, as well as community‐based collection of common recipes and conversion factors. This formative research, as well as the 24‐h dietary recall itself, requires highly skilled and trained data collectors. Training of data collectors for this survey exceeded 2 weeks. The time burden that dietary recall carries means fewer can be done in a day, and high‐maintenance equipment is required (food models, modelling clay, scales, batteries and utensils), all leading to considerably higher costs and longer duration of data collection. In comparison, the survey‐based methods described here require relatively few materials (a graduated cylinder and uncooked rice or other pourable, food‐grade material that will not spoil). Data collectors can be effectively trained in less than 1 day. The indicators required only approximately 5 min to administer in a survey, placing less time burden on respondents and reducing duration and costs of data collection. A comparison of training time, resources/materials and respondent burden for multiple‐pass 24‐h dietary recall and for the indicators described here is shown in Table S5.

There is limited research with which to compare these findings. Previous research on portion size estimates has been limited to recall of specific meals and has been conducted primarily in adolescents or adults and in high‐income countries. Nevertheless, the observation that portion size estimates become less precise with increasing intake is consistent with previous research on portion size estimation using photographs (Ovaskainen et al., 2008; Turconi et al., 2005; Vereecken, Dohogne, Covents, & Maes, 2010). The mean difference between average portions consumed and caregivers' estimate showed a small overestimation of portion size and wide limits of agreement. Wide limits of agreement have been noted in other attempts to validate portion size estimation of specific meals (Flax et al., 2019; Turconi et al., 2005).

It is also important to note that there were some differences in indicators' performance by intervention group. Caregivers in the full intervention group were eligible to receive a feeding bowl and spoon designed to promote age‐specific portion sizes and thick complementary foods, which could influence caregiver awareness and/or report. Indeed, portion size estimates were more strongly correlated with complementary food energy and quantity per episode in the full intervention group. On the contrary, the consistency indicator was not correlated with total complementary food energy intake in the full intervention group.

Complementary food consistency has previously been assessed in surveys, though methods are not well described. To our knowledge, the validity of survey‐based consistency indicators has not been assessed, and little is known about the relationship between consistency and energy intake outside of controlled feeding trials. It has previously been established that the consistency of grain‐based porridges, as measured via a viscometer, is correlated with energy density (Treche & Mbome, 1999). However, viscosity is also impacted by temperature and other ingredients (Black, Pahulu, & Dunn, 2009; Mouquet & Treche, 2001). This could explain why the consistency indicator was only weakly correlated with complementary food energy intake and why it added little value to the identification of children with low complementary food energy intake. The correlation with energy density was somewhat stronger when restricted only to porridges/gruels, likely because the photographs themselves depicted porridges/gruels, but porridges/gruels were not commonly consumed in this sample, while fried and baked dough products were more common. Other possible explanations include that there was insufficient variation in the indicator responses and/or that foods of low energy density were not a driver of low energy intake in this sample. A vast majority (95.5%) of children in this sample consumed complementary foods with more than 0.8 kcal/g, and the median energy density of complementary foods was 1.5 kcal/g. A photographic indicator depicting porridges/gruels of various consistencies may perform better in populations where a greater proportion of the complementary diet is composed of porridges/gruels and/or where there is more variability in energy density of complementary foods.

The AUC of feeding frequency for identifying children with low complementary food energy intake in this sample was similar to those analysed by Dewey, Cohen, Arimond and Ruel (2006). Despite high specificity, minimum meal frequency lacked sensitivity for identifying children with low complementary food energy intake. A dichotomous indicator identifying children with both minimum meal frequency and age‐specific minimum portion size yielded improved sensitivity. It should be noted that these cut‐offs are not intended to act as recommended portion sizes and were chosen based on sensitivity and specificity analyses of portion size in 10‐ml increments. Adding the criterion of having complementary food consistent match photograph 3 or higher caused a large reduction in specificity among infants under 12 months, with only minimal increase in sensitivity.

### 4.1 Strengths and limitations

There are important limitations of this research. First, the sample is limited in its generalizability to older ages and contexts outside of southern Ethiopia. When feeding frequency was originally evaluated as a potential indicator of energy intake from complementary foods, it was done so in 10 data sets from nine countries, and some heterogeneity between sites was observed (Working Group on Infant and Young Child Feeding Indicators, 2006). Therefore, replication of these findings in other populations and older ages is important. No other methods of assessing portion size were tested, but other methods may be more predictive or may yield stronger correlations. It may be useful to develop several potential methods of estimating portion size in order to compare the performance of each. Similarly, the consistency indicator depicts porridges/gruels only, which may have been inappropriate in a population where porridges/gruels comprised less of the overall complementary diet than was expected. Furthermore, both dietary recall and the indicators tested here rely on caregiver recall and self‐report and are susceptible to biases. Future research should assess the performance of these indicators against gold standard dietary assessment methods such as weighed food record and/or doubly labelled water.

It should also be emphasized that the present research does not identify an optimal portion size for infants and young children and is not meant to guide portion size recommendations. Though reducing rates of childhood undernutrition is an important goal, it should not come at the expense of promoting excessive energy intake or overriding an individual child's hunger and fullness cues. The *Guiding Principles for Complementary Feeding of the Breastfed Child* encourages caregivers to respond to a child's feeding cues when determining how much to feed (PAHO/WHO, 2003).

Despite the limitations, there are also strengths of the research presented here. We have evaluated simple indicators for assessing two complementary feeding practices for which no valid indicators have previously been established by comparing them with multiple‐pass 24‐h dietary recalls. We have assessed the correlations in several subgroups, to identify circumstances in which the indicators may lack relative validity. Caregivers' estimate of usual portion size appears to be a valid indicator of complementary food and complementary food energy intake and has added value for identifying children with low complementary food energy intake. The utility of the photographic consistency indicator is less clear. Indicators of complementary food consistency may be more useful in populations where children consume low energy dense foods, particularly porridges/gruels.

Important knowledge gaps remain, however. The performance of these indicators should be assessed in more diverse populations, particularly among older children and in multiple low‐ and middle‐income countries in Africa, Asia and Latin America. Gold standard references, including doubly‐labelled water or weighed food records, should be used to establish indicator validity.

## CONFLICTS OF INTEREST

The authors declare that they have no conflicts of interest.

## CONTRIBUTIONS

ADS, AWG and ECF formulated the research question; ECF and AWG led study design. ECF led the data collection and analysis; ADS and AWG provided input on the analysis and interpretation. ECF wrote the first draft of this manuscript and led manuscript revisions. All authors approved the final version.

## Supporting information


**Figure S1.** Agreement between estimated portion size and average complementary food consumed per meal.
