# Supplementary figures and images for: Portion size and consistency as indicators of complementary food energy intake
Source: Matern Child Nutr. 2021 Feb 2;17(2):e13121. doi: 10.1111/mcn.13121 (PMC7988842; doi:10.1111/mcn.13121)

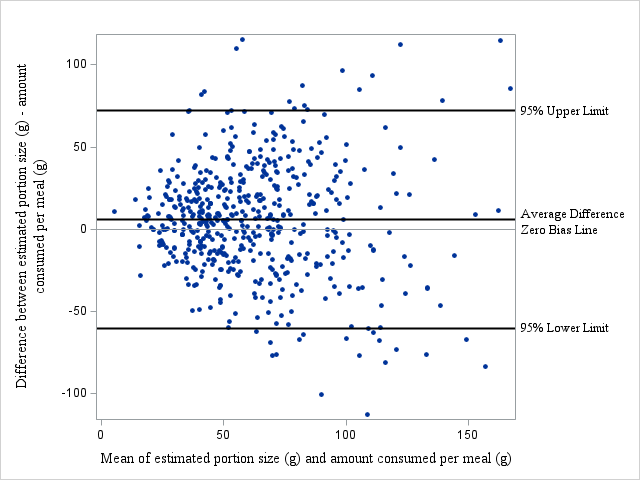

Supplement: Supplementary file 1 — Figure S1. Agreement between estimated portion size and average complementary food consumed per meal. [file MCN-17-e13121-s001.png]
